# Decoding Correlations in Predatory Business Practices and Physicians’ Strategies Against Daily Predatory Emails

**DOI:** 10.7759/cureus.74222

**Published:** 2024-11-22

**Authors:** Alessandro Martinino, Frank Smeenk, Valentina Basile, Juan Pablo Scarano Pereira, Dharmanand Ramnarain, Sjaak Pouwels

**Affiliations:** 1 Surgery, Duke University, Durham, USA; 2 School of Health Professions Education (SHE), Maastricht University, Maastricht, NLD; 3 Internal Medicine, Sanacare Medical Practice, Lugano, CHE; 4 Medicine, Complutense University, Madrid, ESP; 5 Intensive Care Medicine, Saxenburg Medisch Centrum Hardenberg, Tilurg, NLD; 6 Intensive Care Medicine, Elisabeth-Tweesteden Hospital, Tilburg, NLD

**Keywords:** academic spam, electronic mail, predatory journal, survey, time management

## Abstract

Introduction

Predatory journals are marked by inadequate editorial practices and peer review processes, diverging from established global standards in scientific publishing. This article, as a component of the ASGLOS Study, aims to explore the relationship between participant demographics and their experiences with targeted predatory business activities, including their approaches to managing daily predatory emails.

Methods

To collect the personal experiences of physicians’ mailboxes on predatory publishing, a Google Form® survey was designed and disseminated from September 2021 to April 2022.

Results

A total of 978 responses were analyzed from 58 countries around the world. Data underscores a statistically significant association between academic level and email influx (p<0.001). Participants holding a PhD are disproportionately targeted, receiving more than 10 emails/week compared of those without a PhD (p<0.001). Participants with a more prolific publication record, indicated by higher numbers of overall publications, are inclined to directly delete emails. Also, individuals with a higher H Index (p<0.001) are more prone to occasionally responding to these emails.

Conclusion

Our results not only highlight patterns in predatory email reception based on age and academic status but also emphasize the importance of considering academic productivity in understanding the prevalence of predatory solicitations.

## Introduction

Predatory journals represent a concerning and fraudulent phenomenon, significantly impacting scientific research in a destructive manner. Their existence is a widespread concern, as the publication of unscientific and non-peer-reviewed articles holds the potential to adversely influence healthcare and medical decisions [1,2]. Addressing and resolving this issue is crucial to mitigate its harmful consequences.

Predatory journals, marked by inadequate editorial and publishing practices, a primary focus on financial gains, and problematic peer review processes, significantly diverge from established global standards in scientific publishing [3,4]. These journals employ assertive communication techniques, such as persistent phishing emails, and maintain vague organizational standards, deviating sharply from accepted norms worldwide [5,6]. Despite the increasing prevalence of predatory journals, there is currently no universal consensus or comprehensive plan for their global management.

Online resources provide advice to clinicians, but a formal consensus and a united global effort for change are notably absent. This critical gap underscores the urgent necessity for the scientific research community to establish a long-term solution to counter the challenges posed by predatory journals. As part of the ASGLOS Study, this article aims to delve deeper into the relationships among the collected variables, identifying correlations between participants’ demographics and the pursuit of predatory business activities directed at them and exploring how participants’ backgrounds may influence the way to manage and respond to the influx of predatory emails on a daily basis [7]. By providing actionable information and guidance for future initiatives against predatory journals, this study seeks to contribute to the broader goal of addressing and rectifying the issues associated with these problematic publications.

## Materials and methods

Study design and population

To gather the personal experiences of physicians regarding predatory publishing in their mailboxes, two authors (AM, SP) designed an online survey using Google Forms®. The remaining authors collaborated to assemble and edit the survey. The survey consisted of 34 items and was structured into four parts. Part 1 includes demographics (15 questions); Part 2 includes personal experience regarding predatory journals and conferences (12 questions); Part 3 includes personal management regarding predatory emails (6 questions); and Part 4 includes suggestions to solve the problem (1 question). Between September 2021 and April 2022, the survey was distributed across multiple accounts as personal scientific networks of the authors, the Upper Gastrointestinal Surgeon Society (TUGSS) (https://www.tugsglobal.com) social media accounts on Google Group®, Facebook®, Twitter®, LinkedIn®, and the Dutch Societies of Respiratory Medicine, Intensive Care Medicine, and Internal Medicine. 

Descriptive statistics regarding Parts 1, 2, and 3 were previously published [7]. A subsequent paper qualitatively analyses the results obtained from Part 4 [8]. The goal of this project is to perform a comprehensive analysis, delving deeper into the correlation among the variables that have been collected.

Objectives

The primary aim of this study is to explore the correlation between participants' demographics and the weekly rate of receiving predatory emails. By delving into the diverse characteristics of the participants, such as age, academic level, and professional qualifications, we seek to uncover patterns and associations with the volume of predatory solicitations received. 

The secondary objective of this study is to scrutinize participants' demographics in relation to their daily management of predatory emails. By examining how individuals from diverse demographic backgrounds handle and respond to the continuous influx of predatory emails, we aim to uncover patterns and variations in coping strategies.

Together, these investigations aim to provide a comprehensive understanding of how individual demographics may contribute. Indeed, through a meticulous examination of these connections, our study aims to offer valuable insights that can inform targeted interventions and strategies to mitigate the impact of predatory publishing practices on individuals within different demographic groups.

Statistical analysis

Categorical variables were described as proportions of the denominator population and reported as percentages. Continuous variables were tested for normality using the Kolmogorov-Smirnov/Shapiro-Wilk test, and they were presented as median (Interquartile range). Univariate analyses were performed using Pearson Chi-Square for categorical variables and the ANOVA test for continuous variables. The analysis was performed with IBM SPSS Statistics [9]. A p-value <0.05 was considered significant [10].

## Results

A comprehensive analysis was conducted on a total of 978 complete responses with a response rate of 19.9%. The results section is categorized into three parts. Part 1 examines the correlations between participants’ demographics and the pursuit of predatory business activities directed at them, quantified as the weekly volume of received emails. In Part 2, we delve into the correlations between participants’ demographic characteristics and their daily handling of predatory emails. This section aims to explore how various factors may influence the way physicians manage and respond to the influx of predatory emails on a daily basis. Part 3 elucidates the correlation between participants’ demographic characteristics, their familiarity with the "Directory of Open Access Journals," and their inclination to suggest solutions for addressing the associated challenges.

Part 1

Table 1 outlines the correlation with the number of emails per week from predatory journals.

**Table 1:** Correlation with "number of emails/per-week from predatory journals". IF: impact factor, N^o^: number, p-value is considered significant at p<0.001.

Variables	N^o^, emails/week from predatory journals				p-value
	0	1 to 5	5 to 10	>10	
Age, median (IQR)	25 (5)	27 (11)	30 (12)	34 (17)	<0.001
Gender, n (%)	p=0.119	p=0.585	p=0.840	p=0.063	p-value
Male	54 (9.1)	217 (36.5)	145 (24.4)	178 (30)	
Female	48 (12.5)	150 (39.1)	98 (25.5)	88 (22.9)	
Academic qualifications, n (%)	p <0.001	p <0.001	p =0.326	p <0.001	p-value
Medical student	46 (18.3)	118 (46.8)	54 (21.4)	34 (13.5)	
Post-MD training	34 (9.4)	144 (39.9)	96 (26.6)	87 (24.1)	
Attending physician	22 (6)	105 (28.8)	93 (25.5)	145 (39.7)	
Research-oriented, n (%)	p <0.001	p=0.04	p=0.638	p <0.001	p-value
Medicine	41 (9.6)	145 (34)	112 (26.3)	128 (30)	
Surgery	15 (5.5)	101 (37)	66 (24.2)	91 (33)	
Other	46 (16.5)	121 (43.4)	65 (23.3)	47 (16.8)	
MBA, n (%)	p=0.494	p=0.604	p=0.994	p=0.304	p-value
Yes	0	1 (25)	1 (25)	2 (50)	
No	102 (10.5)	366 (37.6)	242 (24.8)	264 (27.1)	
MPH, n (%)	p=0.402	p=0.832	p=0.153	p=0.561	p-value
Yes	0	2 (33.3)	3 (50)	1 (16.7)	
No	102 (10.5)	365 (37.6)	240 (24.7)	265 (27.3)	
PhD, n (%)	p=0.005	p=0.084	p=0.029	p <0.001	p-value
Yes	3 (2.7)	33 (30)	18 (16.4)	56 (50.9)	
No	99 (11.4)	34 (38.5)	225 (25.9)	210 (24.2)	
Type of workplace, n (%)	p=0.625	p=0.789	p=0.072	p=0.073	p-value
Non-academic	32 (9.8)	125 (38.1)	70 (21.3)	101 (30.8)	
Academic	70 (10.8)	242 (37.2)	173 (26.6)	165 (25.4)	
N° overall publications, median (IQR)	2 (4)	5 (9)	8 (21)	20 (67)	<0.001
N° publications as the corresponding author, median (IQR)	0 (1)	2 (3)	3 (7)	8 (27)	<0.001
N° publications as first/last author, median (IQR)	1 (3)	2 (4)	3 (7)	8 (22)	<0.001
H Index, median (IQR)	1 (3)	1 (3)	3 (5)	6 (9)	<0.001
Highest IF accomplished, median (IQR)	2.6 (5.7)	2.7 (3.7)	3.7 (5.2)	6.7 (21.9)	<0.001

In Table 1, data underscores a statistically significant increase in the frequency of predatory journal emails among older individuals (p<0.001). Notably, a striking association exists between academic level and email influx, as medical students demonstrate a lower incidence of receiving emails, while attending physicians bear the brunt, with a majority experiencing over 10 emails weekly (p<0.001). Furthermore, participants holding a PhD are disproportionately targeted, with 50.6% receiving more than 10 emails compared to 24.2% of those without a PhD (p<0.001). The table also describes a compelling link between the volume of predatory emails and participants' academic productivity, as evidenced by heightened rates of overall publications, publications as the corresponding author, publications as first/last author, H-Index, and highest impact factor achieved (all p<0.001). This nuanced portrayal of email reception sheds light on the intersection of age, academic status, and predatory journal targeting, offering valuable insights into the scholarly landscape. 

Table 2 delineates the correlation with the number of emails per week from predatory conferences and presents findings similar to those in Table 1. The variables "gender" and "type of workplace" also demonstrate statistical significance in relation to the number of emails received.

**Table 2:** Correlation with "number of emails/per-week from predatory conferences". IF: impact factor, N^o^: number, p-value is considered significant at p<0.001.

Variables	N° emails/per-week from predatory conferences				p-value
	0	0 to 5	5 to 10	>10	
Age, median (IQR)	25 (6)	29 (12)	31 (14)	32 (15)	<0.001
Gender, n (%)	p=0.007	p=0.094	p=0.844	p=0.451	p-value
Male	94 (15.8)	235 (39.6)	128 (21.5)	137 (23.1)	
Female	86 (22.4)	133 (34.6)	87 (22.7)	78 (20.3)	
Academic qualifications, n (%)	p <0.001	p=0.04	p=0.08	p=0.352	p-value
Medical student	78 (31)	89 (35.3)	51 (20.2)	34 (13.5)	
Post-MD training	64 (17.7)	154 (42.7)	70 (29.4)	73 (20.2)	
Attending physician	38 (10.4)	125 (34.2)	94 (25.8)	108 (29.6)	
Research oriented, n (%)	p <0.001	p=0.018	p=0.110	p=0.352	p-value
Medicine	71 (16.7)	149 (35)	106 (24.9)	100 (23.5)	
Surgery	31 (11.4)	122 (44.7)	58 (21.2)	62 (22.7)	
Other	78 (28)	97 (34.8)	51 (18.3)	53 (19)	
MBA, n (%)	p=0.341	p=0.609	p=0.175	p=0.287	p-value
Yes	0	2 (50)	2 (50)	0	
No	180 (18.5)	366 (37.6)	213 (21.9)	215 (22.1)	
MPH, n (%)	p=0.912	p=0.141	p=0.752	p=0.192	p-value
Yes	1 (16.7)	4 (66.7)	1 (16.7)	0	
No	179 (18.4)	364 (37.4)	214 (22)	215 (22.1)	
PhD, n (%)	p=0.003	p=0.451	p=0.004	p=0.307	p-value
Yes	9 (8.2)	45 (40.9)	36 (32.7)	20 (18.2)	
No	171 (19.7)	323 (37.2)	179 (20.6)	195 (22.5)	
Type of workplace, n (%)	p=0.102	p=0.230	p 0.021	p=0.015	p-value
Non-Academic	51 (15.5)	132 (40.2)	58 (17.7)	87 (26.5)	
Academic	129 (19.8)	236 (36.3)	157 (24.2)	128 (19.7)	
N° overall publications, median (IQR)	3 (4)	6 (16)	13 (30)	14 (48)	<0.001
N° publications as corresponding author, median (IQR)	1 (2)	2 (5)	5 (12)	5 (18)	<0.001
N° publications as first/last author, median (IQR)	1 (3)	3 (6)	5.5 (12)	7 (16)	<0.001
H Index, median (IQR)	1 (2)	2 (4)	4 (6)	4 (8)	<0.001
Highest IF accomplished, median (IQR)	2.3 (3.8)	3.5 (5.2)	4.3 (7.3)	5.7 (11.9)	0.02

Part 2

As described in Table 3, having a PhD stands out as a statistically significant variable (p<0.001) when it comes to capturing the reader's attention; indeed, a higher percentage of individuals with a PhD (46.4%) exhibit a tendency to directly delete emails compared to those without a PhD (26.5%). Moreover, individuals with a more prolific publication record, indicated by higher numbers of overall publications (p=0.01), publications as the first/last author (p=0.023), and a higher achieved highest IF (p<0.001), are inclined to directly delete emails.

**Table 3:** Correlation with, "how do you read each email"? IF: impact factor, p-value is considered significant at p<0.001.

Variables	How do you read each email?			p-value
	Carefully	Quickly	Directly delete	
Age, median (IQR)	30 (16)	29 (12)	30 (17)	0.009
Gender, n (%)	p=0.065	p=0.171	p=0.452	p-value
Male	140 (23.5)	276 (46.4)	178 (29.9)	
Female	84 (21.8)	197 (51.3)	103 (26.8)	
Academic qualifications, n (%)	p=0.920	p=0.355	p=0.267	p-value
Medical student	60 (23.8)	124 (49.2)	68 (27)	
Post-MD training	81 (22.4)	183 (50.7)	97 (26.9)	
Attending physician	83 (22.7)	166 (45.5)	116 (31.8)	
Research-oriented, n (%)	p=0.003	p=0.212	p=0.156	p-value
Medicine	102 (23.9)	196 (46)	128 (30)	
Surgery	44 (16.1)	144 (52.7)	85 (31.1)	
Other	78 (28)	133 (47.7)	68 (24.4)	
MBA, n (%)	p=0.196	p=0.349	p=0.869	p-value
Yes	2 (50)	1 (25)	1 (25)	
No	222 (22.8)	472 (48.5)	280 (28.7)	
MPH, n (%)	p=0.715	p=0.936	p=0.803	p-value
Yes	1 (16.7)	3 (50)	2 (33.3)	
No	223 (22.9)	470 (48.4)	279 (28.7)	
PhD, n (%)	p <0.001	p=0.292	p <0.001	p-value
Yes	11 (10)	48 (43.6)	51 (46.4)	
No	213 (24.5)	425 (49)	230 (26.5)	
Type of workplace, n (%)	p=0.017	p <0.001	p=0.056	p-value
Non-academic	90 (27.4)	131 (39.9)	107 (32.6)	
Academic	134 (20.6)	342 (52.6)	174 (26.8)	
N° overall publications, median (IQR)	8 (22)	7 (16)	10 (30)	0.01
N° publications as the corresponding author, median (IQR)	3 (13)	2 (6)	4 (9)	0.113
N° publications as first/last author, median (IQR)	3 (11)	3 (6)	4 (13)	0.023
H Index, median (IQR)	3 (5)	2 (4)	3 (7)	0.099
Highest IF accomplished, median (IQR)	3.8 (5.1)	3.3 (5.6)	5.2 (12.6)	<0.001

Age is a factor influencing response patterns, with older individuals more likely to refrain from replying to emails (p=0.045) (Table 4). Interestingly, those with a PhD exhibit a higher frequency of replies (73.6%), yet no one consistently replies to these emails, as reflected by a significant p-value of 0.002. Furthermore, individuals with a higher H Index (p<0.001) and those who have achieved a higher Highest IF (p=0.004) are more prone to occasionally responding to these emails.

**Table 4:** Correlation with “do you ever reply to the email for more information?” IF: impact factor, p-value is considered significant at p<0.001.

Variables	Do you ever reply to the email for more information?			p-value
	Never	Sometimes	Always	
Age, median (IQR)	31 (14)	28 (14)	28 (10)	0.045
Gender, n (%)	p=0.059	p=0.198	p=0.524	p-value
Male	226 (38)	323 (54.3)	45 (7.7)	
Female	160 (41.6)	197 (51.4)	27 (7)	
Academic qualifications, n (%)	p=0.607	p=0.721	p=0.451	p-value
Medical student	93 (36.9)	137 (54.4)	22 (8.7)	
Post-MD training	144 (39.9)	195 (54)	22 (6.1)	
Attending physician	149 (40.8)	188 (51.5)	28 (7.7)	
Research oriented, n (%)	p=0.217	p=0.067	p=0.112	p-value
Medicine	173 (40.6)	228 (53.5)	25 (5.9)	
Surgery	96 (35.2)	158 (57.9)	19 (7)	
Other	117 (42)	134 (48)	28 (10)	
MBA, n (%)	p=0.553	p=0.381	p=0.572	p-value
Yes	1 (25)	3 (75)	0	
No	385 (39.5)	517 (53.1)	72 (7.4)	
MPH, n (%)	p=0.758	p=0.506	p=0.489	p-value
Yes	2 (33.3)	4 (66.7)	0	
No	384 (39.5)	516 (53.1)	72 (7.4)	
PhD, n (%)	p=0.003	p <0.001	p=0.002	p-value
Yes	29 (26.4)	81 (73.6)	0	
No	357 (41.1)	439 (50.6)	72 (8.3)	
Type of workplace, n (%)	p=0.623	p=0.957	p=0.414	p-value
Non-academic	133 (40.5)	174 (53)	21 (6.4)	
Academic	253 (38.9)	346 (53.2)	51 (7.8)	
N° overall publications, median (IQR)	8 (18)	8 (26)	5 (10)	0.006
N° publications as corresponding author, median (IQR)	4 (9)	3 (9)	2 (4)	0.081
N° publications as first/last author, median (IQR)	3 (8)	3 (10)	2 (4)	0.005
H Index, median (IQR)	2 (4)	3 (7)	1 (3)	< 0.001
Highest IF accomplished, median (IQR)	3.6 (5.1)	4.6 (7.7)	2.5 (4.8)	0.004

In Table 5, age emerged as a non-statistically significant factor influencing the likelihood of participants unsubscribing from the predatory email lists. Notably, individuals with a PhD (p=0.002) and MBA (p=0.030) exhibited a lower frequency of unsubscribing, with fewer than 25% of emails. On the contrary, those with the highest number of publications (p=0.031), particularly as the first/last author (p=0.025) and corresponding author (p=0.036), demonstrated a higher tendency to unsubscribe to more than 75% of the emails.

**Table 5:** Correlation with “do you unsubscribe from these email distribution lists?” and “do you block these email addresses?” IF: impact factor, p-value is considered significant at p<0.001.

Variables	Do you unsubscribe from these email distribution lists?				p-value
	< 25%	25% - 50%	50% - 75%	> 75%	
Age, median (IQR)	29 (16)	30 (10)	29 (14)	29 (14)	0.245
Gender, n (%)	p=0.679	p=0.264	p=0.394	p=0.436	p-value
Male	163 (27.4)	144 (24.2)	68 (11.4)	219 (36.8)	
Female	100 (26)	82 (21.3)	49 (12.7)	153 (39.8)	
Academic qualifications, n (%)	p=0.628	p=0.007	p=0.121	p=0.401	p-value
Medical student	72 (28.6)	47 (18.7)	28 (11.1)	105 (41.7)	
Post-MD training	91 (25.2)	103 (28.5)	35 (9.7)	132 (36.6)	
Attending physician	100 (27.4)	76 (20.8)	53 (14.5)	136 (37.3)	
Research-oriented, n (%)	p=0.464	p=0.456	p=0.103	p=0.231	p-value
Medicine	121 (28.4)	99 (23.2)	54 (12.7)	152 (35.7)	
Surgery	66 (24.2)	69 (25.3)	23 (8.4)	115 (42.1)	
Other	76 (27.2)	58 (20.8)	39 (14)	106 (38)	
MBA, n (%)	p=0.030	p=0.272	p=0.462	p=0.588	p-value
Yes	3 (75)	0	0	1 (25)	
No	260 (26.7)	226 (23.2)	116 (11.9)	372 (38.2)	
MPH, n (%)	p=0.721	p=0.117	p=0.367	p=0.277	p-value
Yes	2 (33.3)	3 (50)	0	1 (16.7)	
No	261 (26.9)	223 (22.9)	116 (11.9)	372 (38.3)	
PhD, n (%)	p=0.002	p=0.001	p=0.114	p=0.293	p-value
Yes	43 (39.1)	12 (10.9)	8 (7.3)	47 (42.7)	
No	220 (25.3)	214 (24.7)	108 (12.4)	326 (37.6)	
Type of workplace, n (%)	p=0.521	p=0.319	p=0.984	p=0.770	p-value
Non-Academic	84 (25.6)	82 (25)	39 (11.9)	123 (37.5)	
Academic	179 (27.5)	144 (63.7)	77 (11.8)	250 (38.5)	
N° overall publications, median (IQR)	6 (21)	7 (15)	7 (12)	10 (32)	0.031
N° publications as corresponding author, median (IQR)	2 (7)	3 (6)	3 (5)	4 (11)	0.036
N° publications as first/last author, median (IQR)	3 (9)	3 (6)	3 (6)	5 (13)	0.025
H Index, median (IQR)	2 (4)	2 (4)	2 (6)	3 (6)	0.264
Highest IF accomplished, median (IQR)	4.3 (7.7)	3.4 (5.4)	3 (5.4)	4.5 (7)	<0.001

Older individuals displayed a propensity to block between 50-75% of these email senders (p=0.036), while younger individuals, particularly medical students (p=0.034), were inclined to block fewer senders (less than 25%) (Table 6). Interestingly, individuals with a PhD, although less likely to unsubscribe, were found to block the highest percentage of senders, exceeding 75% (p=0.005).

**Table 6:** Correlation with “do you block these email addresses?” IF: impact factor, p-value is considered significant at p<0.001.

Variables	Do you block these email addresses?”				p-value
	<25%	25%-50%	50% -75%	>75%	
Age, median (IQR)	28 (13)	29 (11)	31 (14)	30 (16)	0.036
Gender, n (%)	p=0.510	p=0.509	p=0.208	p=0.460	p-value
Male	199 (33.5)	138 (23.2)	66 (11.1)	191 (32.2)	
Female	136 (35.4)	83 (21.6)	35 (9.2)	130 (33.8)	
Academic qualifications, n (%)	p=0.034	p=0.708	p=0.334	p=0.061	p-value
Medical student	103 (40.9)	61 (24.2)	20 (7.9)	68 (27)	
Post-MD training	118 (32.7)	82 (22.7)	39 (10.8)	122 (33.8)	
Attending physician	114 (31.2)	78 (21.4)	42 (11.5)	131 (35.9)	
Research-oriented, n (%)	p=0.325	p=0.438	p=0.193	p=0.087	p-value
Medicine	137 (32.2)	88 (20.7)	51 (12)	150 (35.2)	
Surgery	93 (34.1)	65 (23.8)	21 (7.7)	94 (34.4)	
Other	105 (37.6)	68 (24.4)	29 (10.4)	77 (27.6)	
MBA, n (%)	p=0.005	p=0.279	p=0.496	p=0.161	p-value
Yes	4 (100)	0	0	0	
No	331 (34)	221 (22.7)	101 (10.4)	321 (33)	
MPH, n (%)	p=0.962	p=0.528	p=0.609	p=0.398	p-value
Yes	2 (33.3)	2 (33.3)	1 (16.7)	1 (16.7)	
No	333 (34.3)	219 (22.5)	100 (10.3)	320 (32.9)	
PhD, n (%)	p = 0.178	p < 0.001	p = 0.075	p = 0.005	p-value
Yes	44 (40)	11 (10)	6 (5.5)	49 (44.5)	
No	291 (33.5)	210 (24.2)	95 (10.9)	272 (31.3)	
Type of workplace, n (%)	p=0.632	p=0.265	p=0.977	p=0.598	p-value
Non-Academic	109 (32.5)	81 (36.7)	34 (33.7)	104 (32.4)	
Academic	226 (67.5)	140 (63.3)	67 (66.3)	271 (67.6)	
N° overall publications, median (IQR)	6 (19)	8 (15)	7 (18)	11 (32)	0.023
N° publications as corresponding author, median (IQR)	2 (7)	3 (6)	3 (8)	4 (12)	0.068
N° publications as first/last author, median (IQR)	2 (8)	3 (5)	3 (8)	5 (14)	0.053
H Index, median (IQR)	2 (5)	2 (5)	2 (5)	3 (8)	0.169
Highest IF accomplished, median (IQR)	4 (5.7)	3.5 (5.2)	3.3 (6.5)	4.5 (8)	0.851

Examining the data outlined in Table 7, it becomes apparent that age does not yield statistical significance in the ability of individuals to differentiate a true journal invitation from a predatory spam. However, intriguingly, there exists a statistical significance indicating that male participants tend to find this distinction easier than their female counterparts. Furthermore, authors with a higher number of publications demonstrate an enhanced proficiency in this regard, with a statistically significant association observed (p=0.002).

**Table 7:** Correlation with “how hard do you find it to distinguish an actual invitation from spam?” IF: impact factor, p-value is considered significant at p<0.001.

Variables	How do you find it to distinguish an actual invitation from spam?					p-value
	1 (easy)	2	3	4	5 (hard)	
Age, median (IQR)	28 (13)	30 (17)	30 (13)	29.5 (12)	35 (13)	0.316
Gender, n (%)	p=0.002	p=0.051	p=0.360	p=0.072	p <0.001	p-value
Male	151 (25.4)	144 (24.2)	151 (25.4)	122 (20.5)	26 (4.4)	
Female	73 (19)	74 (19.3)	104 (27.1)	94 (24.5)	39 (10.2)	
Academic qualifications, n (%)	p=0.558	p=0.306	p=0.541	p=0.240	p=0.521	p-value
Medical student	53 (21)	53 (21)	65 (25.8)	63 (25)	18 (7.1)	
Post-MD training	89 (24.7)	74 (20.5)	101 (28)	70 (19.4)	27 (7.5)	
Attending physician	82 (22.5)	91 (24.9)	89 (24.4)	83 (22.7)	20 (5.5)	
Research oriented, n (%)	p=0.028	p=0.626	p=0.622	p=0.007	p=0.814	p-value
Medicine	99 (23.2)	96 (22.5)	117 (27.5)	85 (20)	29 (6.8)	
Surgery	75 (27.4)	65 (23.8)	66 (24.2)	51 (18.7)	16 (5.9)	
Other	50 (17.9)	57 (20.4)	72 (25.8)	80 (28.7)	20 (7.2)	
MBA, n (%)	p=0.196	p=0.182	p=0.234	p=0.286	p=0.593	p-value
Yes	2 (50)	2 (50)	0	0	0	
No	222 (22.8)	216 (22.2)	255 (26.2)	216 (22.2)	65 (6.7)	
MPH, n (%)	p=0.715	p <0.001	p=0.145	p=0.191	p=0.512	p-value
Yes	1 (16.7)	5 (83.3)	0	0	0	
No	223 (22.9)	213 (21.9)	255 (26.2)	216 (22.2)	65 (6.7)	
PhD, n (%)	p=0.846	p=0.183	p=0.220	p=0.006	p=0.899	p-value
Yes	26 (23.6)	30 (27.3)	34 (30.9)	13 (11.8)	7 (6.4)	
No	198 (22.8)	188 (21.7)	221 (25.5)	203 (23.4)	58 (6.7)	
Type of workplace, n (%)	p=0.103	p=0.985	p=0.697	p=0.927	p <0.001	p-value
Non-academic	65 (19.8)	73 (22.3)	83 (25.3)	73 (22.3)	34 (10.43)	
Academic	159 (24.5)	145 (22.3)	172 (26.5)	143 (22)	31 (4.8)	
N° overall publications, median (IQR)	8 (24)	12 (24)	7 (22)	7 (14)	7 (28)	0.002
N° publications as the corresponding author, median (IQR)	3 (9)	5 (11)	3 (8)	2 (6)	3 (10)	0.047
N° publications as first/last author, median (IQR)	3 (11)	4 (11)	2 (9)	3 (6)	3 (9)	0.011
H Index, median (IQR)	3 (6)	4 (7)	2 (6)	2 (5)	2 (4)	0.005
Highest IF accomplished, median (IQR)	4.5 (7.5)	4.5 (5.9)	3.7 (5.3)	2.9 (6)	4.7 (6.8)	0.072

Table 8 reveals a notable pattern where older individuals and those with a greater number of publications tend to cluster within the range of 10-20 emails before achieving a discerning ability to differentiate between genuine invitations and spam. In contrast, medical students constitute the majority of participants who remain uncertain in this regard (p<0.001).

**Table 8:** Correlation with “how long did it take to learn to discriminate this type of email from actual invitations?” IF: impact factor, p-value is considered significant at p<0.001.

Variables	How long did it take to learn to discriminate this type of email from actual invitations?					p-value
	Right away	< 5 emails	5-10	10-20	Still unsure	
Age, median (IQR)	27 (9)	30 (11)	35 (17)	37 (16)	28.5 (18)	<0.001
Gender, n (%)	p=0.741	p=0.122	p=0.157	p=0.336	p <0.001	p-value
Male	126 (21.2)	335 (56.4)	60 (10.1)	22 (3.7)	51 (8.6)	
Female	81 (21.1)	193 (50.3)	30 (7.8)	19 (4.9)	61 (15.9)	
Academic qualifications, n (%)	p=0.304	p=0.480	p <0.001	p=0.002	p <0.001	p-value
Medical student	59 (23.4)	132 (52.4)	15 (6)	1 (0.4)	45 (17.9)	
Post-MD training	80 (22.2)	204 (56.5)	24 (6.6)	18 (5)	35 (9.7)	
Attending physician	68 (18.6)	192 (52.6)	51 (14)	22 (6)	32 (8.8)	
Research oriented, n (%)	p=0.531	p=0.921	p=0.502	p=0.056	p=0.014	p-value
Medicine	88 (20.7)	230 (54)	43 (10.1)	23 (5.4)	42 (9.9)	
Surgery	64 (23.4)	145 (53.1)	26 (9.5)	13 (4.8)	25 (9.2)	
Other	55 (19.7)	153 (54.8)	21 (7.5)	5 (1.8)	45 (16.1)	
MBA, n (%)	p=0.851	p=0.244	p=0.005	p=0.675	p=0.471	p-value
Yes	1 (25)	1 (25)	2 (50)	0	0	
No	206 (21.1)	527 (54.1)	88 (9)	41 (4.2)	112 (11.5)	
MPH, n (%)	p=0.464	p=0.532	p=0.434	p=0.607	p=0.377	p-value
Yes	2 (33.3)	4 (66.7)	0	0	0	
No	205 (21.1)	524 (53.9)	90 (9.3)	41 (4.2)	112 (11.5)	
PhD, n (%)	p=0.944	p=0.021	p=0.002	p=0.087	p=0.849	p-value
Yes	23 (20.9)	48 (43.6)	19 (17.3)	8 (7.3)	12 (10.9)	
No	184 (21.2)	480 (55.3)	71 (8.2)	33 (3.8)	100 (11.5)	
Type of workplace, n (%)	p=0.118	p=0.691	p=0.173	p=0.272	p=0.586	p-value
Non-Academic	60 (18.3)	180 (54.9)	36 (11)	17 (5.2)	35 (10.7)	
Academic	147 (22.6)	348 (53.5)	54 (8.3)	24 (3.7)	77 (11.8)	
N° overall publications, median (IQR)	7 (17)	7 (17)	20 (69)	35 (66)	5 (16)	<0.001
N° publications as corresponding author, median (IQR)	2 (5)	3 (7)	7 (28)	15 (35)	2 (5)	<0.001
N° publications as first/last author, median (IQR)	3 (8)	3 (6)	8 (28)	20 (35)	2 (8)	<0.001
H Index, median (IQR)	2 (5)	2 (4)	4 (10)	7 (11)	2 (4)	<0.001
Highest IF accomplished, median (IQR)	3.9 (5.6)	4.1 (5.4)	4.5 (14.4)	6.4 (21.6)	3.2 (5.6)	0.235

Part 3

The data presented in Table 9 highlights individuals with a PhD (p=0.048) and those with a greater number of papers as corresponding authors (p=0.044) demonstrate a heightened familiarity with the Directory of Open Access Journals (DOAJ).

**Table 9:** Correlation with “have you heard of the Directory of Open Access Journals (DOAJ)?” IF: impact factor, p-value is considered significant at p<0.001.

Variables	Have you heard of the DOAJ?		p-value
	Yes	No	
Age, median (IQR)	29.5 (12)	30 (16)	0.192
Gender, n (%)			0.731
Male	413 (69.5)	181 (30.5)	
Female	263 (68.5)	121(31.5)	
Academic qualifications, n (%)			0.775
Medical student	178 (70.6)	74 (29.4)	
Post-MD training	250 (69.3)	111 (30.7)	
Attending physician	248 (67.9)	117 (32.1)	
Research oriented to, n (%)			0.824
Medicine	290 (68.1)	136 (31.9)	
Surgery	191 (70)	82 (30)	
Other	195 (69.9)	84 (30.1)	
MBA, n (%)			0.799
Yes	3 (75)	1 (25)	
No	673 (69.1)	301 (30.9)	
MPH, n (%)			0.896
Yes	4 (66.7)	2 (33.3)	
No	672 (69.1)	300 (30.9)	
PhD, n (%)			0.048
Yes	67 (60.9)	43 (39.1)	
No	609 (70.2)	259 (29.8)	
Type of workplace, n (%)			0.154
Non-Academic	217 (66.2)	111 (33.8)	
Academic	459 (70.6)	191(29.4)	
N° overall publications, median (IQR)	9 (21)	4.5 (21)	0.064
N° publications as corresponding author, median (IQR)	3 (9)	2 (8)	0.044
N° publications as first/last author, median (IQR)	3 (8)	2 (8)	0.265
H Index, median (IQR)	3 (5)	1 (5)	0.062
Highest IF accomplished, median (IQR)	4.1 (6.2)	4.1 (6.1)	0.319

Also, individuals who presented suggestions for managing the identified problem encompass older individuals (p<0.001), participants within the surgery domain (p=0.023), individuals holding an MPH degree (p=0.025), those with a Ph.D. (p<0.001), and participants with a higher research output as outlined in Table 10.

**Table 10:** Correlation with “do you have any suggestions to manage the problem?” IF: impact factor, p-value is considered significant at p<0.001.

Variables	Do you have any suggestions to manage the problem?		p-value
	Yes	No	
Age, median (IQR)	33.5 (18)	29 (13)	<0.001
Gender, n (%)			0.082
Male	107 (18)	487 (82)	
Female	53 (13.8)	331 (86.2)	
Academic qualifications, n (%)			0.1
Medical student	32 (12.7)	220 (87.3)	
Post-MD training	58 (16.1)	303 (83.9)	
Attending physician	70 (19.2)	295 (80.8)	
Research-oriented, n (%)			0.023
Medicine	74 (17.4)	352 (82.6)	
Surgery	54 (19.4)	219 (80.2)	
Other	32 (11.5)	247 (88.5)	
MBA, n (%)			0.64
Yes	1(25)	3 (75)	
No	159 (16.3)	815 (83.7)	
MPH, n (%)			0.025
Yes	3 (50)	3 (50)	
No	157 (16.2)	815 (83.8)	
PhD, n (%)			<0.001
Yes	42 (38.2)	68 (61.8)	
No	118 (13.6)	750 (86.4)	
Type of workplace, n (%)			0.328
Non-Academic	59 (18)	269 (82)	
Academic	101 (15.5)	549 (84.5)	
N° overall publications, median (IQR)	18.5 (72)	7 (17)	<0.001
N° publications as corresponding author, median (IQR)	7.50 (29)	3 (6)	<0.001
N° publications as first/last author, median (IQR)	9 (29)	3 (7)	<0.001
H Index, median (IQR)	4 (11)	2 (5)	<0.001
Highest IF accomplished, median (IQR)	6.5 (28.7)	3.6 (5.6)	<0.001

## Discussion

To our knowledge, the ASGLOS online survey stands out as one of the most extensive and inclusive collections of information regarding predatory journals and conferences worldwide. This survey involved a cross-sectional evaluation of the email solicitations that researchers received throughout various stages of their careers. In our first published study, we presented a descriptive analysis of our results, encompassing an assessment of 978 complete responses [7]. The participants exhibited a broad spectrum in their academic paths, ranging from medical students (25.8%) to tenured professors in their specific fields. This diversity addresses a limitation noted in previous studies on similar subjects, where medical students were typically underrepresented [11]. While the first article provided a descriptive account of the variables collected in the survey, the current study aims to delve more profoundly into the relationships among the gathered variables, discerning correlations between participants' demographics and the predatory business activities targeted at them [7]. Additionally, it explores how diverse participants’ backgrounds may impact the strategies employed to manage predatory emails on a daily basis. Summarizing, the results of the ASGLOS online global survey emphasize the problem of academic email spam and underscore the importance of organizing training sessions to increase awareness about predatory publishing for individuals involved in scientific research.

Understanding why researchers choose to publish in predatory journals is crucial for creating effective interventions to deter future submissions. Academic life has been a subject of considerable changes, especially in recent years. The advent of the internet and social media has significantly contributed to equality in science and fear of deprivation within academic society. “Publish or perish” is a multidimensional problem and needs perspectives and solutions from diverse related aspects. Indeed, it is a new phenomenon and, like other newly emerged entities, needs brand-new solutions and deserves more attention [12]. The “publish or perish” mantra has misled the ultimate goal of academia as centers for teaching and shifted away the focus of universities and scientific institutions from education to competition [13]. “Publication at all costs” is a direct product of the “publish or perish” environment. This paves the way for fraud, misconduct, or other unethical behaviors [14,15].

Concerning our primary objective of this study, the findings on the frequency of predatory journal emails offer a comprehensive and nuanced insight into the intricacies of this phenomenon's dynamics. The statistically significant associations with various academic indicators, including overall publications, corresponding authorship, first/last authorship, H-Index, and highest Impact Factor achieved, suggest that predatory emails may be more prevalent in environments with higher academic output. This raises questions about why certain academic groups are more targeted by predatory solicitations and how awareness campaigns can be tailored to address specific needs within different academic levels. This is also in line with the contrast between medical students, who experience a lower incidence of predatory emails, and attending physicians, who bear a substantial burden, with a majority receiving over 10 emails weekly, suggesting a differential targeting strategy based on the academic output. Also, the disproportionate targeting of participants with a PhD, where over 50% receive more than 10 emails compared to a lower percentage among those without a PhD, highlights the need to understand why individuals with higher academic qualifications are more heavily solicited. Exploring whether predatory publishers specifically target individuals with advanced degrees or if this group is more active in academic networks that attract such solicitations could provide valuable insights.

This study's secondary objective is to analyze the demographic characteristics of participants concerning their daily handling of predatory emails. By exploring how people from various demographic groups manage the constant stream of predatory emails, we seek to reveal different approaches and trends in coping methods. Indeed, we found it interesting to investigate further the main profiles of predatory journals’ targets and the factors that support their tendency to be attracted by these fraudulent requests, such as the level of self-control, the level of prior knowledge in the field, the ability to discern between true and false information, and the characteristic of their academic networks [16].

Identifying predatory journals prior to submission is crucial, as reputable journals are unlikely to send the authors random emails soliciting articles [17]. In spite of the sparse literature on identifying predatory email solicitations, there are some red flags common to predatory journals that librarians and researchers can apply when scrutinizing unsolicited manuscript solicitations. These red flags include spamming, short deadlines for manuscript submission, non-personalized or erroneous salutations, and a scope that doesn't match the researchers' field [18]. In our finding, there is a statistically significant difference based on academic qualification regarding how long it took to learn to discriminate this type of email from actual invitations. Compared to attending physicians and post-MD training individuals, medical students think they still have difficulty learning to discriminate this type of email from actual invitations. This aligns with a previous study by Alamri et al., reporting a general lack of awareness regarding predatory journals among medical students [19]. Nevertheless, it's crucial to acknowledge that the results might be biased since medical students rarely receive real invitations and may have been more exposed to them through their mentors. Interestingly, we observed that males, in comparison to females, express a higher level of confidence in their ability to distinguish between a genuine invitation and spam. This suggests a gender-based disparity in perceived self-reported confidence, a concept that has already been extensively studied [20-22]. Also, professionals working in non-academic workplaces find it more difficult to distinguish a spam email from a genuine invitation compared to those working in academic environments. Overall, these results are in accordance with a general hypothesis that the inexperience of neophyte researchers and students can play a role in discriminating predatory journals [23,24].

According to our data collection, individuals in non-academic workplaces also exhibit a carefully reading approach towards emails from predatory journals in contrast to those in academic environments. As discussed before, this subset of participants also faced greater challenges in discerning predatory emails and may be more susceptible to falling into traps. Apart from the fact that their lack of confidence on the topic may necessitate more attention in reading these emails, we can also hypothesize that “victims” of predatory journals may be aware that their past decisions led to unfavorable outcomes. With such experiences, they will delay decision-making, which may reduce emotional involvement and will gain extra time for a more careful examination [16]. In contrast, individuals with a higher level of expertise, as evidenced by a greater number of overall publications, publications as the first/last author, and the highest impact factor achieved, are inclined to choose direct email deletion. 

By exploring how people from various groups manage the constant stream of predatory emails, we seek to reveal different approaches and trends in coping methods. Based on our gathered data, younger individuals are more prone to consistently request additional information, whereas older individuals typically opt to block the sender of such emails more frequently. Also, individuals with a PhD sometimes ask for more information more frequently than those without a PhD. This could be ascribed to the fact that individuals with a PhD are often immersed in the academic sphere and face pressures related to ongoing commitments and delivery [25]. However, researchers must acknowledge that despite the pressure to produce scientific work, they must consider the ethical consequences of sacrificing academic integrity and critical evaluation by utilizing publication platforms prioritizing monetary gain over scholarly merit [26,27].

Limitations and strengths

While our research demonstrated robustness, it is essential to acknowledge certain limitations. Initially, all parameters relied on self-reporting, introducing the potential for recall bias inherent systematic error stemming from participants inaccurately recalling past experiences or omitting details. This condition is susceptible to influences from subsequent events and experiences, impacting the accuracy and volume of memories. Additionally, our survey questionnaire was implemented without prior pre-testing or validation, although it underwent thorough scrutiny by an expert panel of consultants. Lastly, the large volume of comparisons made in this research raises the potential for false positives.

Nevertheless, our online survey featured one of the most extensive aggregates of respondents worldwide, contributing to the promotion of awareness among medical students and professionals. Our findings' implications highlight the importance of offering career-oriented guidance in academia and arranging training sessions tailored for researchers to augment their awareness of predatory publishing.

## Conclusions

In conclusion, these results not only highlight patterns in predatory email reception based on age and academic status but also emphasize the importance of considering academic productivity in understanding the prevalence of predatory solicitations. The findings provide a foundation for targeted interventions and awareness campaigns that address specific vulnerabilities within different demographic and academic groups, ultimately contributing to a more informed and resilient scholarly community.
